# Insights into Nodal T-Follicular Helper Cell lymphomas and Peripheral T-Cell Lymphomas, Not Otherwise Specified, in Slovenian Patients: Mutational Landscape, Clinicopathological Characteristics, and Outcomes

**DOI:** 10.1007/s00277-025-06709-z

**Published:** 2025-11-13

**Authors:** Eva Erzar, Vanesa Sindi-Ivanova, Stefan Dirnhofer, Lučka Boltežar, Janja Ocvirk, Veronika Kloboves Prevodnik, Alexandar Tzankov, Gorana Gašljević

**Affiliations:** 1 Department of Cytopathology, Institute of Oncology Ljubljana, Ljubljana, Slovenia; 2 Faculty of Medicine, University of Ljubljana, Ljubljana, Slovenia; 3 Pathology, Institute of Medical Genetics and Pathology, University Hospital Basel, University of Basel, Basel, Switzerland; 4 Department of Medical Oncology, Institute of Oncology Ljubljana, Ljubljana, Slovenia; 5 Faculty of Medicine, University of Maribor, Maribor, Slovenia; 6 Department of Pathology, Institute of Oncology Ljubljana, Ljubljana, Slovenia

**Keywords:** nodal T-follicular helper cell lymphomas (nTFHL), peripheral T-cell lymphoma, not otherwise specified (PTCL-NOS), mutational landscape, survival analysis, prognostic factors

## Abstract

Nodal T-follicular helper (TFH) cell lymphomas (nTFHLs) are the most common mature nodal T-cell lymphomas (nTCL) in Europe, characterized by an aggressive course, poor prognosis, and recurrent expression of TFH markers. Their mutational landscape and the impact of these mutations on survival outcomes remain underexplored. In this study, we characterized nTCL features in a Slovenian cohort, comprising 91 nTFHL, 9 peripheral TCL, not otherwise specified (PTCL-NOS), and 8 composite lymphomas [co-occurrence of nTFHL, angioimmunoblastic type (nTFHL-AI) with monoclonal B-cell proliferation], with a median follow up of 23 months. Using immunohistochemistry, clonality testing, and high-throughput sequencing with a lymphoma panel targeting 172 genes, we analyzed their mutational landscape and examined the mutational impact on survival outcomes. The mutational analysis uncovered *TET2* (43%), *RHOA* (26%), *IDH2* (9%), *PLCG1* (8%), and *DNMT3A* (6%) as the most commonly mutated genes. *RHOA* mutations correlated with *TET2*, *IDH2*, and *DNMT3A* mutations, and CD10 expression was linked to *TET2* mutations. Multiple mutations, high International Prognostic Index, progressive disease after first-line treatment, and the absence of autologous stem cell transplantation (ASCT) were independent predictors for shorter survival. To date, this study is one of the largest nTCL series, confirming that nTFHLs outnumber PTCL-NOS (92% vs. 8%). Our findings underscore the complex role of genetic factors in nTCL’s clinical behaviour and emphasize the importance of ASCT. We also highlight the need for prospective clinical trials, which explore tailored therapeutic interventions, such as hypomethylating agents or IDH inhibitors, for improving survival in specific genetic contexts.

**Supplementary Information:**

The online version contains supplementary material available at 10.1007/s00277-025-06709-z.

## Introduction

Nodal T-cell lymphomas (nTCLs) are rare, heterogeneous malignancies characterized by nodal presentation, poor prognosis, and a challenging differential diagnosis. Recent advancements in mutational profiling have improved our understanding of nTCL pathogenesis, contributing to refined classification, increased diagnostic accuracy, and identification of potential targeted therapeutic strategies.

Examining the mutational landscape of nTCL subtypes is particularly valuable for diagnostic purposes when biopsy material is limited and/or morphology is atypical. While similar mutations occur across multiple nTCL subtypes, their distribution varies, with certain alterations being more prevalent in specific subtypes. Nodal T-follicular helper cell lymphomas (nTFHL), which display a T-follicular helper (TFH)-related phenotype, frequently exhibit mutations in epigenetic regulators such as *TET2*, *DNMT3A*, and *IDH2* [1–8]. Additionally, mutations leading to T-cell activation, such as *RHOA*, are also prevalent in nTFHL [5, 9–11], but occur less frequently in other nTCL subtypes [3–8, 12]. The *IDH2*^R172X^ variant appears to be specific to nTFHL, angioimmunoblastic type (nTFHL-AI), occurring in 20–45% of cases, and co-occurring with *TET2* mutations in 70–90% of instances [8, 11, 13–15]. The *RHOA*^G17V^ variant accounts for more than two-thirds of all *RHOA* mutations in nTFHL-AI and frequently co-occurs with *TET2*, *DNMT3A*, and *IDH2* mutations [4, 9–11, 13, 14]. Less frequent mutations, observed across several nTCLs, affect co-stimulatory or T-cell receptor (TCR) signalling genes, including *CD28*,* PLCG1*,* CARD11*, and *CTNNB1* [4, 11, 16–18]. Beyond diagnostic utility, mutations in epigenetic regulators in nTFHLs have important clinical implications. Such cases respond better to histone deacetylase and DNA methyltransferase inhibitors compared with non-TFH nTCLs [19, 20]. Furthermore, certain individual mutations or co-mutation patterns have been associated with survival outcomes in nTCLs [21–27], although their prognostic impact remains largely unexplored.

In peripheral T-cell lymphoma, not otherwise specified (PTCL-NOS), alterations in *TP53*, *PRDM1*, *PTEN*, *CDKN2A/B*, *STAT3*, and *MYC* are associated with the more aggressive PTCL-GATA3 subtype. In contrast, PTCL-TBX21, which harbours mutations in *TET1*, *TET3*, and *DNMT3A*, is thought to have better prognosis [25, 28]. However, Tyler et al. demonstrated that PTCL-TBX21 patients with *DNMT3A* mutations have similarly poor outcomes to those with PTCL-GATA3 [22].

Most studies on nTCLs have been conducted in Western Europe [21, 29], the USA [25, 30–33], and Asia [34–36], focusing on subtype frequencies, their clinicopathological, immunophenotypical, and genetic characteristics. However, data on the incidence, prognostic markers, mutational landscape, and survival outcomes of nTFHLs and PTCL-NOS in South-Eastern Europe, remain scarce, with no publications from this region. Our study addresses this gap by analyzing these aspects in 108 Slovenian patients diagnosed with nTFHL and PTCL-NOS.

## Materials and methods

### Study population

This study included 108 Slovenian patients diagnosed with nTFHL, PTCL-NOS, or composite lymphomas (CL, i.e., co-occurrence of nTFHL-AI and B-cell lymphoma) between 2007 and 2022, all of whom received treatment at the Institute of Oncology Ljubljana (IOL), Slovenia. Their histological samples (lymphadenectomies) were retrieved from the IOL archives. Data on patient age, clinical stage, laboratory parameters, IPI score, treatment protocols, and outcomes were obtained from the electronic medical records in the hospital information system.

### Central review of tumour samples

This retrospective analysis included a re-evaluation of all initial diagnoses by three expert hematopathologists (AT, SD and GG). The review comprised morphological and immunophenotypical examination of archived haematoxylin and eosin (H&E) and immunohistochemical (IHC) slides, supplemented with additional IHC staining and PCR-based analyses of B- and T-cell clonality (BIOMED-2) [37]. Following the review, all selected samples were re-labelled and some were re-classified according to the WHO 5^th^ edition [38] and ICC 2022 [39] into one of the following categories: nTFHL, angioimmunoblastic type (nTFHL-AI); nTFHL, follicular type (nTFHL-F); nTFHL, not otherwise specified (nTFHL-NOS); PTCL-NOS; or CL (nTFHL-AI combined with DLBCL or MZL).

### TMA construction

Tissue microarrays (TMAs) were constructed by sampling two morphologically representative cores of a 2-mm diameter in a 40-core format array into a new paraffin block using a TMA workstation (Beecher Instruments, USA) according to previously described procedures [40].

### Immunohistochemistry

From formalin-fixed paraffin-embedded (FFPE) whole-tissue samples or TMAs, 1 μm thick sections were sliced. Automated IHC slide staining was performed at the OIL and the Institute of Medical Genetics and Pathology, University Hospital Basel on BenchMark Ultra (Roche/Ventana Medical Systems, Tucson, AZ, USA) using OptiView detection kits (Roche). IHC was performed according to the standard operating procedure (SOP) of the institutes. Details of all used antibodies and SOPs are described in Table S1.

### Molecular analysis

#### Clonality testing

The results of the clonality analysis of lymphocyte populations by PCR were obtained from the hospital information system (OIL), since clonality was routinely determined for diagnosis. The BIOMED-2 clonality assays-ABI Fluorescence Detection method (IdentiClone; InVivo Scribe Technologies, San Diego, CA, USA) was used according to the manufacturer’s instructions. The determination of T-cell lymphocyte clonality was conducted for samples lacking this information.

#### DNA extraction for next-generation sequencing (NGS)

DNA was isolated from 10 μm thick sliced untreated FFPE tissue samples by using the MagMAX™ FFPE DNA/RNA Ultra Kit (#A31881, Thermo Fisher Scientific, Waltham, MA, USA). Only cases with a tumor cell content (TCC) > 15% were deemed eligible for NGS.

***NGS*** Ninety-nine nPTCL samples had sufficient DNA for NGS, performed using the IonAmpliSeq™ customized, validated and ISO15189-accredited lymphoma panel comprising 4716 amplicons (size range: 125–175) covering 172 genes (Thermo Fisher Scientific, Waltham, MA, USA). Library preparation, chip loading, variant annotation, and variant classification were conducted precisely as previously described [41].

### Statistical analysis

Descriptive statistics were used to summarize the clinical data. Statistical analyses were conducted using SPSS software (version 28.0.1.0, IBM, Armonk, NY, USA) and RStudio. The R package “BiocManager (maftools)” was used to generate an oncoplot with mutational hierarchy. Pearson’s Chi-square or Fisher’s exact test was applied for correlations between categorical variables, with Bonferroni correction for multiple comparisons. The Kaplan-Meier method (with log-rank test) was used to generate survival curves and assess differences in overall survival (OS) and progression-free survival (PFS). OS was defined as the time from histopathologic diagnosis to death from any cause or end of follow up, and PFS as the time from histopathologic diagnosis to disease progression, death, or end of follow up. The Cox proportional hazards model was used to evaluate the independent impact of potential prognostic factors on survival outcomes. Variables with *p* ≤ 0.10 in univariate analyses were included in the multivariate model. Potential confounding factors that were already part of other variables, such as ECOG PS and LDH (both components of the IPI score), were excluded. Differences with *p* < 0.05 were considered statistically significant. Patient vital status was obtained from the Cancer Registry of the Republic of Slovenia on April 15, 2024. Median survival was expressed in months.

## Results

### Review of tumour samples

**Fig. 1:**
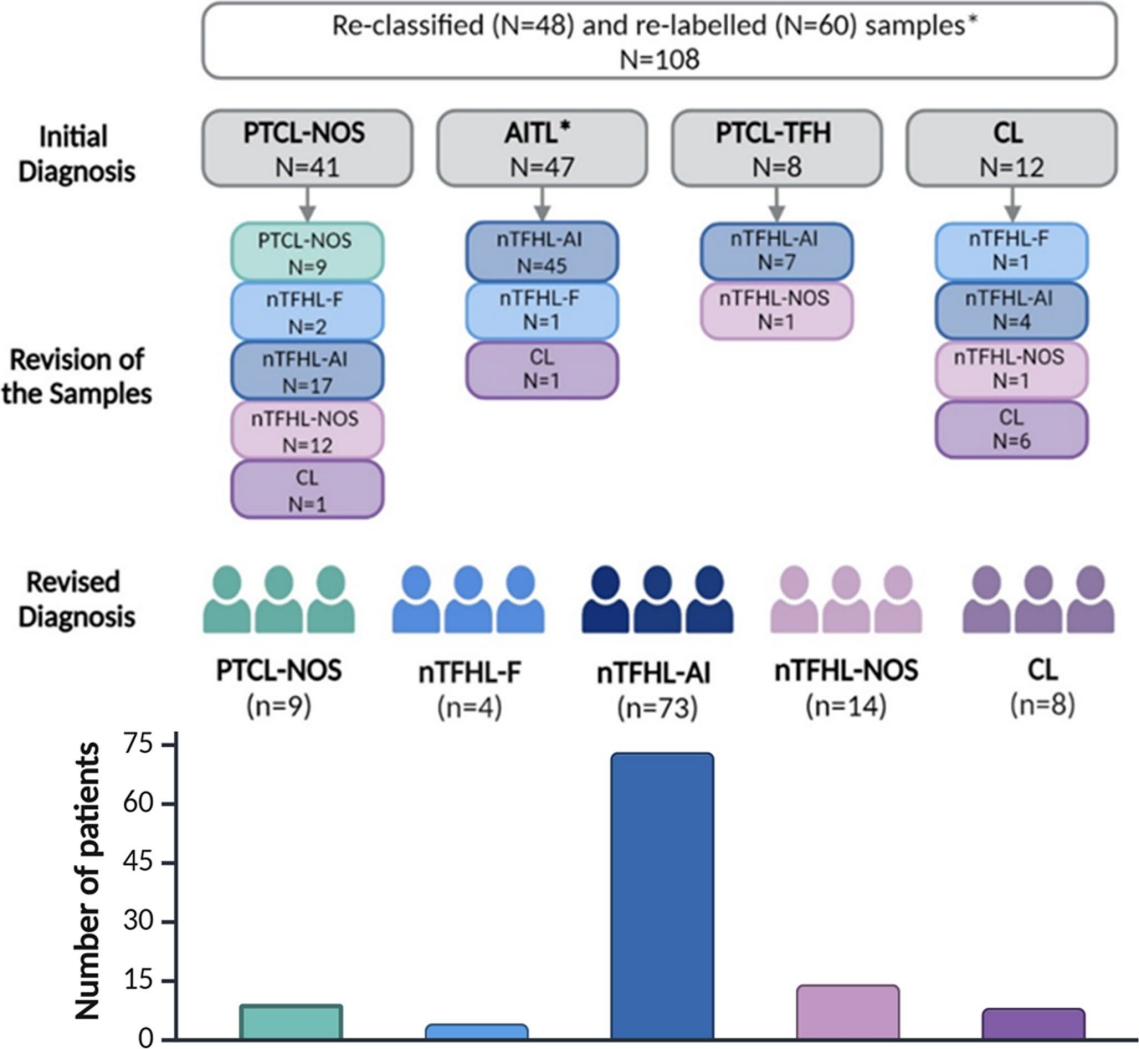
Re-classification and re-labelling of patient samples based on diagnostic revision. * AITL (now re-labelled to nTFHL-AI)

After diagnostic revision, the 108 nTCL patients were categorized into 5 entities: PTCL-NOS, nTFHL-F, nTFHL-AI, nTFHL-NOS, and CL (Fig. 1). Cases diagnosed as nTFHL-AI were further sub-classified into patterns: pattern 1 (*n* = 7), pattern 2 (*n* = 34), and pattern 3 (*n* = 30). In 2 nTFHL-AI samples, the pattern could not be determined due to copious necrosis. In certain equivocal cases, specific mutational findings with the expression of 2 TFH markers prompted re-evaluation of a few PTCL-NOS diagnoses. Consequently, three patients (P53, P93, and P105) were re-classified as nTFHL based on the following information (Supplementary Materials, Table S2).

### Clinical characteristics

The clinical characteristics of the study cohort are summarized in Table 1. The median patient age was 69 years (range 26–87). At diagnosis, ¾ of the patients (*n* = 75; 69%) had a good Eastern Cooperative Oncology Group (ECOG) performance status (PS) of 0–1. The majority (*n* = 100; 93%) presented with advanced disease (Ann Arbor stage III–IV), and 63% were classified in the high-intermediate or high-risk categories of the International Prognostic Index (IPI).

**Table 1:** Clinical characteristics with administered first-line treatment

Characteristics	Overall *N* (%)
**Number of patients**	108 (100)
**Diagnosis**	
nTFHL-AI	73 (68)
nTFHL-NOS	14 (13)
nTFHL-F	4 (4)
CL	8 (7)
PTCL-NOS	9 (8)
**Gender**	
Male	58 (54)
Female	50 (46)
**Age at diagnosis**	
> 60 years	85 (79)
≤ 60	23 (21)
**Ann Arbor stage**	
I	1 (1)
II	7 (6)
III	26 (24)
IV	74 (69)
**B-symptoms**	
No	36 (33)
Yes	72 (67)
**Raised serum LDH level**	
No	47 (44)
Yes	59 (64)
Not available/unknown	2 (2)
**ECOG performance status**	
0	37 (34)
1	38 (35)
2	20 (19)
3	5 (5)
4	8 (7)
**IPI-risk groups**	
Low (0–1)	13 (12)
Low-intermediate (2)	25 (23)
High-intermediate (3)	41 (38)
High (4–5)	27 (25)
Not available/unknown	2 (2)
**First-line treatment**	
CHOP/CHOP-like	20 (19)
COP/modified COP/other low dose treatments	62 (57)
Other	18 (17)
None	8 (7)
**Response to first-line treatment**	
Complete response (CR)	31 (29)
Partial response (PR)	26 (24)
Stable disease (SD)	6 (6)
Progressive disease (PD)	28 (26)
Not applicable (no first-line treatment)	8 (7)
Not available/unknown	9 (8)
**State of the patient**	
Alive	27 (25)
Dead	81 (75)

PTCL-NOS: peripheral T-cell lymphoma, not otherwise specified; nTFHL-NOS: nodal T-follicular helper cell lymphoma, not otherwise specified; nTFHL-F: nodal T-follicular helper cell lymphoma, follicular type; nTFHL-AI: nodal T-follicular helper cell lymphoma, angioimmunoblastic type; CL: composite lymphoma; LDH: lactate dehydrogenase; ECOG: Eastern Cooperative Oncology Group; IPI: International Prognostic Index; COP: Cyclophosphamide, Oncovin (Vincristine) and Prednisone; CHOP: Cyclophosphamide, Hydroxydaunorubicin (Doxorubicin/Adriamycin), Oncovin (Vincristine) and Prednisone

### Treatment type and response rates

**Fig. 2:**
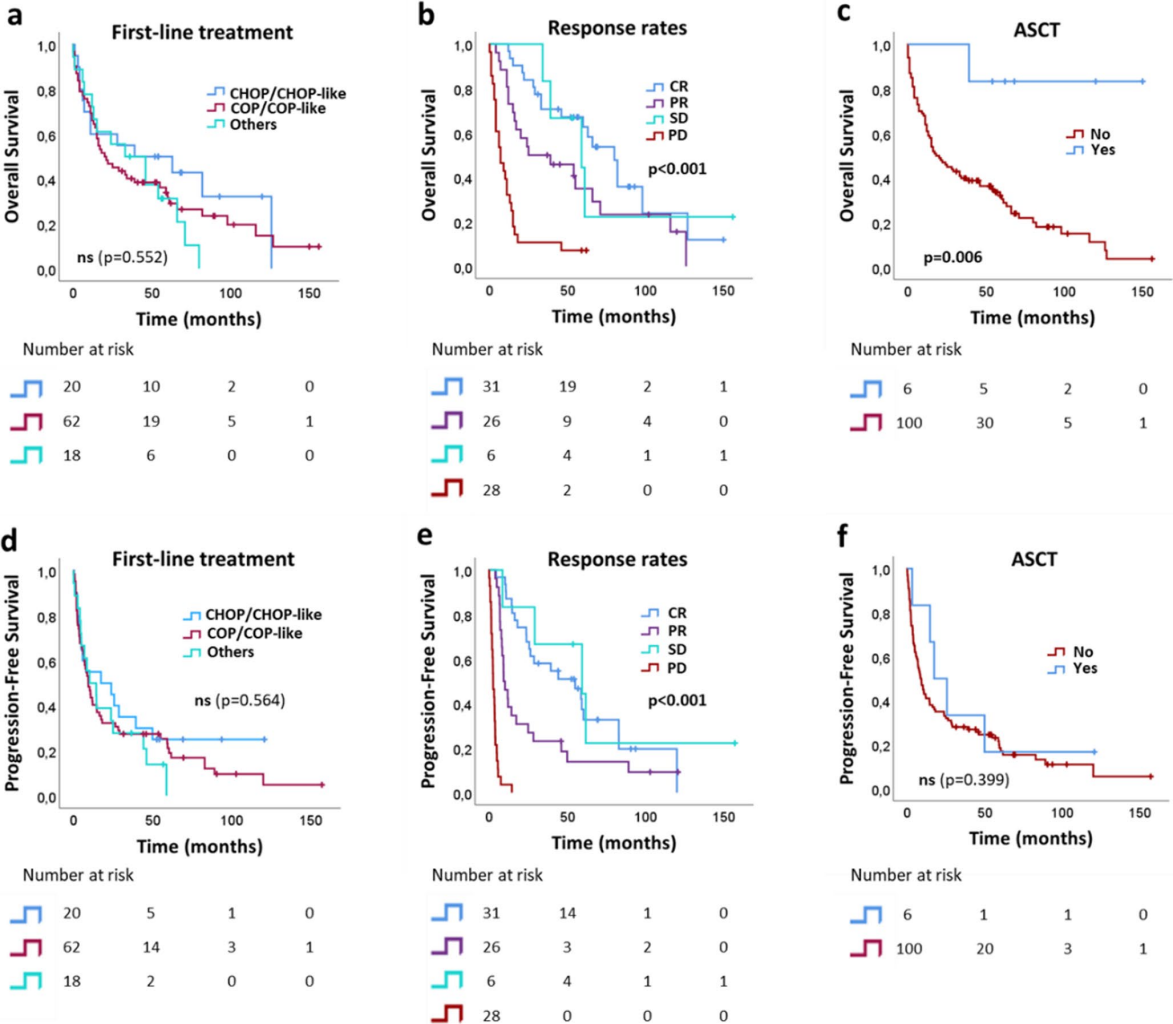
Kaplan-Meier plots showing overall survival and progression-free survival of nTCL patients based on (**a**, **d**) first-line treatment, (**b**, **e**) response rates to first-line treatment, and (**c**, **f**) ASCT as consolidation therapy. CHOP: Cyclophosphamide, Hydroxydaunorubicin (Doxorubicin or Adriamycin), Oncovin (Vincristine) and Prednisone; COP: Cyclophosphamide, Oncovin (Vincristine) and Prednisone; NA: not applicable (no first-line treatment); CR: complete response; PR: partial response; SD: stable disease; PD: progressive disease; ASCT: autologous stem cell transplantation; ns: not significant

First-line treatment types and their response rates are displayed in Table 1. Eight patients received autologous (hematopoietic) stem cell transplantation (ASCT) as consolidation therapy. The overall response rate (ORR) to COP/COP-like or other low-dose regimens was 51.6% (*n* = 32/62), with 27.4% achieving complete remission (CR) and 24.2% partial remission (PR). For CHOP/CHOP-like regimens, the ORR was 65% (*n* = 13/20), with a CR of 40% and a PR of 25%. No significant difference in OS and PFS was observed between patients receiving different first-line treatments (*p* = 0.522; Fig. 2a and d; Table 2). However, OS and PFS differed significantly according to response to first-line treatment (*p* < 0.001; Fig. 2b and e; Table 2). Patients who received ASCT as consolidation therapy had longer OS than those who did not (*p* = 0.006; Fig. 2c; Table 2), but there was no association with PFS (Fig. 2f; Table 2). In the multivariate Cox proportional hazards model for OS, ASCT emerged as an independent favourable prognostic factor, whereas progressive disease (PD) after first-line treatment was an independent adverse factor (Table 3). Patients with PD had a 7.682-fold higher risk of death (95% CI 3.419–17.262, *p* = < 0.001) compared with patients achieving CR (Table 3).

**Table 2:** Evaluation of clinical characteristic and common mutations as potential prognostic factors for overall survival and progression-free survival in nTCLs was performed using univariate analysis (Log-rank test). The factors in bold were included in the subsequent multivariate analysis (Cox proportional hazards model) based on the criteria described in the methods section

	Univariate analysis			
	**OS**		**PFS**	
Variables	**Chi-Square**	**P value**	**Chi-Square**	**P value**
Gender	0.523	0.469	0.447	0.504
Age ≥ 60	0.524	0.469	0.428	0.513
Advanced Ann Arbor stage (III-IV)	1.714	0.190	2.421	0.120
B-symptoms	0.723	0.395	0.932	0.334
Elevated LDH	8.009	0.005	5.754	0.016
ECOG ≤ 1	21.171	< 0.001	15.133	< 0.001
IPI-risk groups	52.887	**< 0.001**	36.182	**< 0.001**
First-line treatment	1.299	0.522	1.146	0.564
Response rates	50.851	**< 0.001**	112.973	**< 0.001**
ASCT	7.593	**0.006**	0.713	0.399
*TET2*	2.204	0.138	0.516	0.472
*RHOA*	0.473	0.492	0.727	0.394
*IDH2*	1.365	0.243	0.095	0.758
*TET2/RHOA*	0.002	0.963	0.287	0.592
*TET2/IDH2*	1.235	0.266	0.212	0.645
Number of mutations	7.255	**0.027**	6.575	**0.037**

LDH: lactate dehydrogenase; ECOG: Eastern Cooperative Oncology Group; IPI: International Prognostic Index; ASCT: autologous stem cell transplantation; *TET2*: Tet Methylcytosine Dioxygenase 2; *RHOA*: Ras Homolog Family Member A; *IDH2*: Isocitrate Dehydrogenase (NADP (+)) 2; OS: overall survival; PFS: progression-free survival

**Table 3:** Results of multivariate analysis (Cox proportional hazards model) identifying independent prognostic factors for overall survival and progression-free survival in nTCLs.

	Multivariate analysis			
	**OS**		**PFS**	
Variables	**HR (95% CI)**	**P value**	**HR (95% CI)**	**P value**
IPI-risk groups		< 0.001		0.006
Low (0–1)	-	-	-	-
Low-intermediate (2)	1.025 (0.369–2.843)	0.963	0.817 (0.339–1.968)	0.652
High-intermediate (3)	1.263 (0.470–3.394)	0.644	1.145 (0.524–2.504)	0.734
High (4–5)	5.832 (1.964–17.315)	0.001	3.429 (1.351–8.703)	0.010
Response rates		< 0.001		< 0.001
CR	**-**	-	-	-
PR	1.692 (0.817–3.506)	0.157	1.494 (0.775–2.881)	0.230
SD	0.540 (0.147–1.989)	0.354	0.421 (0.116–1.529)	0.188
PD	7.682 (3.419–17.262)	< 0.001	22.403 (9.770–51.368.770.368)	< 0.001
ASCT	0.085 (0.011–0.649)	-	-	-
Number of mutations		0.045		0.040
0	**-**	-	-	-
1	1.634 (0.752–3.551)	0.215	2.351 (1.207–4.580)	0.012
≥ 2	2.232 (1.182–4.214)	0.013	1.481 (0.821–2.671)	0.191

LDH: lactate dehydrogenase; ECOG: Eastern Cooperative Oncology Group; IPI: International Prognostic Index; CR: complete response; PR: partial response; SD: stable disease; PD: progressive disease; ASCT: autologous stem cell transplantation; OS: overall survival; PFS: progression-free survival

### Mutational landscape of nTFHL, PTCL-NOS, and CLs

**Fig. 3:**
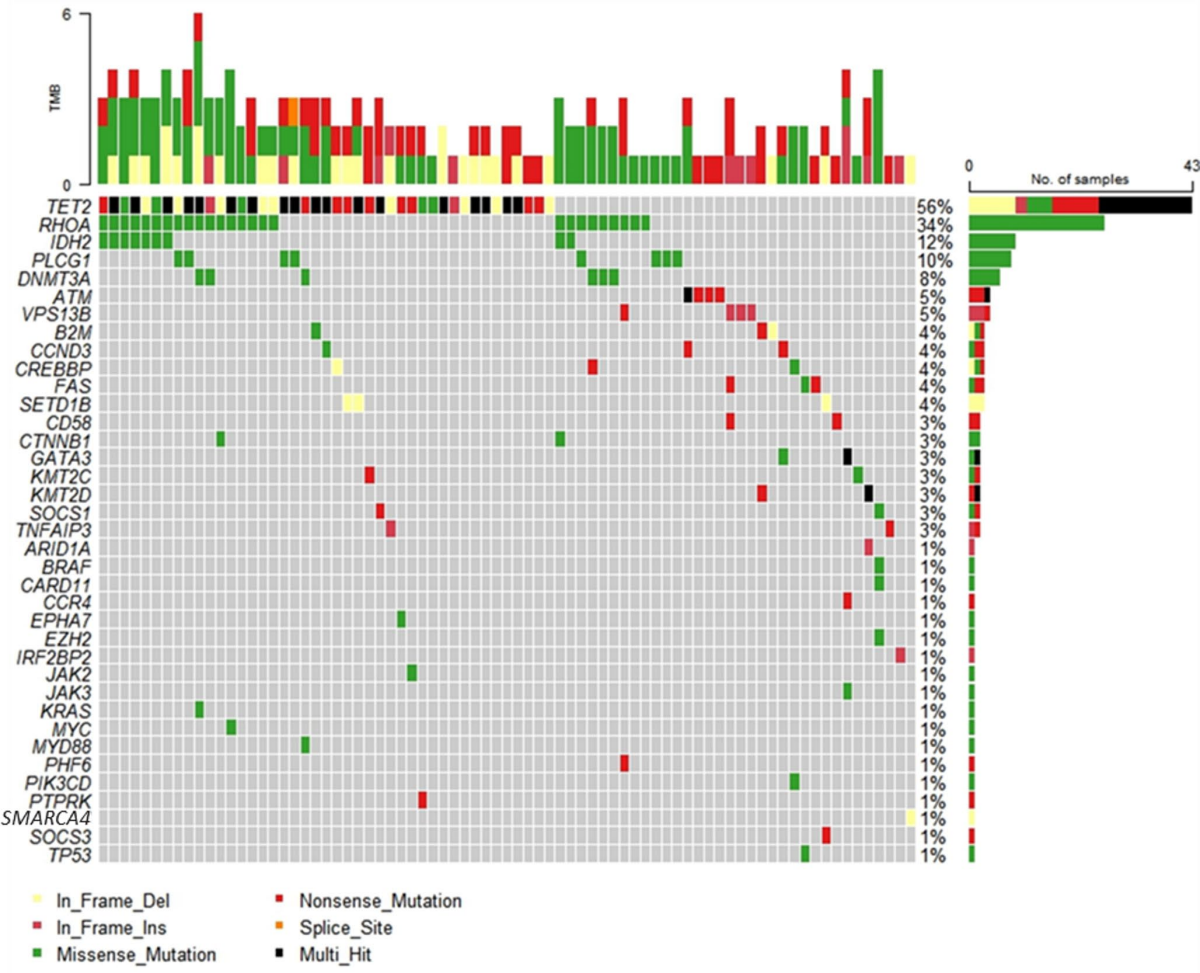
The mutational landscape detected by the targeted NGS lymphoma panel is shown for 77 patients diagnosed with nTFHL-AI, nTFHL-NOS, nTFHL-F, PTCL-NOS, and CL (nTFHL-AI combined with monoclonal B-cell proliferation) who harboured mutations. The heatmap displays all distinct genetic mutations identified by NGS, excluding samples without any detected pathogenic, likely pathogenic mutations, or variants of unknown significance (*n* = 22). Each column represents an individual patient, while each row corresponds to a mutated gene. Percentages indicate the proportion of patients with mutations in each detected gene. Block colours indicate mutation types, with black blocks representing multiple alterations in the same gene. Bar graphs above the heatmap show the number of mutations in each sample. TMB = Tumour mutation burden

Of the 108 patient samples, 99 were sequenced; 9 were excluded due to DNA fragmentation. Among the 99 interpretable cases, 53 harboured pathogenic (P) or likely pathogenic (LP) variants, 24 had only variants of uncertain significance (VUS) (Supplementary Materials, Table S3), and 22 had no detectable mutations. All results and comparisons in this section refer to the total number of mutations (including P, LP variants, and VUS). Twenty-four patients had 1 mutation, while 53 had ≥ 2 different mutations, most often in different genes. *TET2* mutations were the most common (*n* = 43, 43%), including 17 double and 1 triple mutation. These were followed by *RHOA* mutations (*n* = 26, 26%), with the *RHOA*^G17V^ variant accounting for 88.5% (*n* = 23/26); the other detected variants were E47K, R5Q and R5W. *IDH2* was mutated in 9% of cases, *PLCG1* in 8%, and *DNMT3A* in 6% – all found exclusively in nTFHLs. Overall, the cohort showed a heterogeneous mutational profile (Fig. 3). Only *IDH2* mutations were subtype-specific, occurring solely in nTFHL-AI patients (12.3%). *TET2*,* GATA3*,* KMT2D*, and *ATM* were the only genes with ≥ 2 simultaneous mutations in the same gene, indicated by black squares in Fig. 3. In *TET2*, multiple mutations occurred in 18 samples, all of which were nTFHL-AI pattern 2 (*n* = 7), pattern 3 (*n* = 6), CL (*n* = 3), or nTFHL-NOS (*n* = 2). *TET2* and *RHOA* mutations were more frequent in nTFHL-AI (50% and 34%, respectively) than in other nTFHL subtypes (24% and 18%). In contrast, *PLCG1* (9% vs. 12%) and *DNMT3A* (4% vs. 18%) mutations were slightly less frequent. In CL, *TET2* mutations were more common (63%), with no detected *RHOA* or *DNMT3A* mutations.

Significant co-occurrence patterns were observed: *RHOA* mutations with *IDH2 (p* < 0.001), *TET2* (*p* = 0.009), or *DNMT3A* (*p* = 0.004); and *TET2* mutations with *IDH2* (*p* = 0.038) but not with *DNMT3A* (*p* = 1.00). *RHOA* mutations co-occurred with *IDH2* in 9 samples, *TET2* in 17, and *DNMT3A* in 5. All *IDH2* mutations (*n* = 9) were *IDH2*^R172^ variants, which co-occurred with *RHOA*^G17V^ mutations in all cases, and with *TET2* mutations in 7 (78%). A total of 169 mutations were identified across all panel genes. Most were missense mutations (48%), followed by nonsense mutations (28%) and frameshift-INDELs (22%). The remaining 2% were non-frameshift deletions or splice site mutations. All mutations in *RHOA*, *IDH2*, *DNMT3A*, and *PLCG1* were missense. In contrast, *TET2* mutations included nonsense (43%), frameshift-INDELs (39%), missense (15%), and other types (3%).

### Association of TFH markers and clinical characteristics with mutations

CD10 expression was significantly associated with *TET2* (*p* = 0.003) mutations, while no such association was found for the other TFH markers. Detailed results of associations between TFH markers and frequently mutated genes in nTCLs are provided in Supplementary Materials, Table S4.

Main clinical parameters showed no association with *TET2* or *RHOA* mutations (Supplementary Materials, Table S5). Although *TET2* mutations were initially associated with B-symptoms (*p* = 0.034) and lymphoma subtype (*p* = 0.045), these associations lost significance after Bonferroni correction for multiple comparisons.

### Impact of clinical characteristics and mutations on survival

**Fig. 4:**
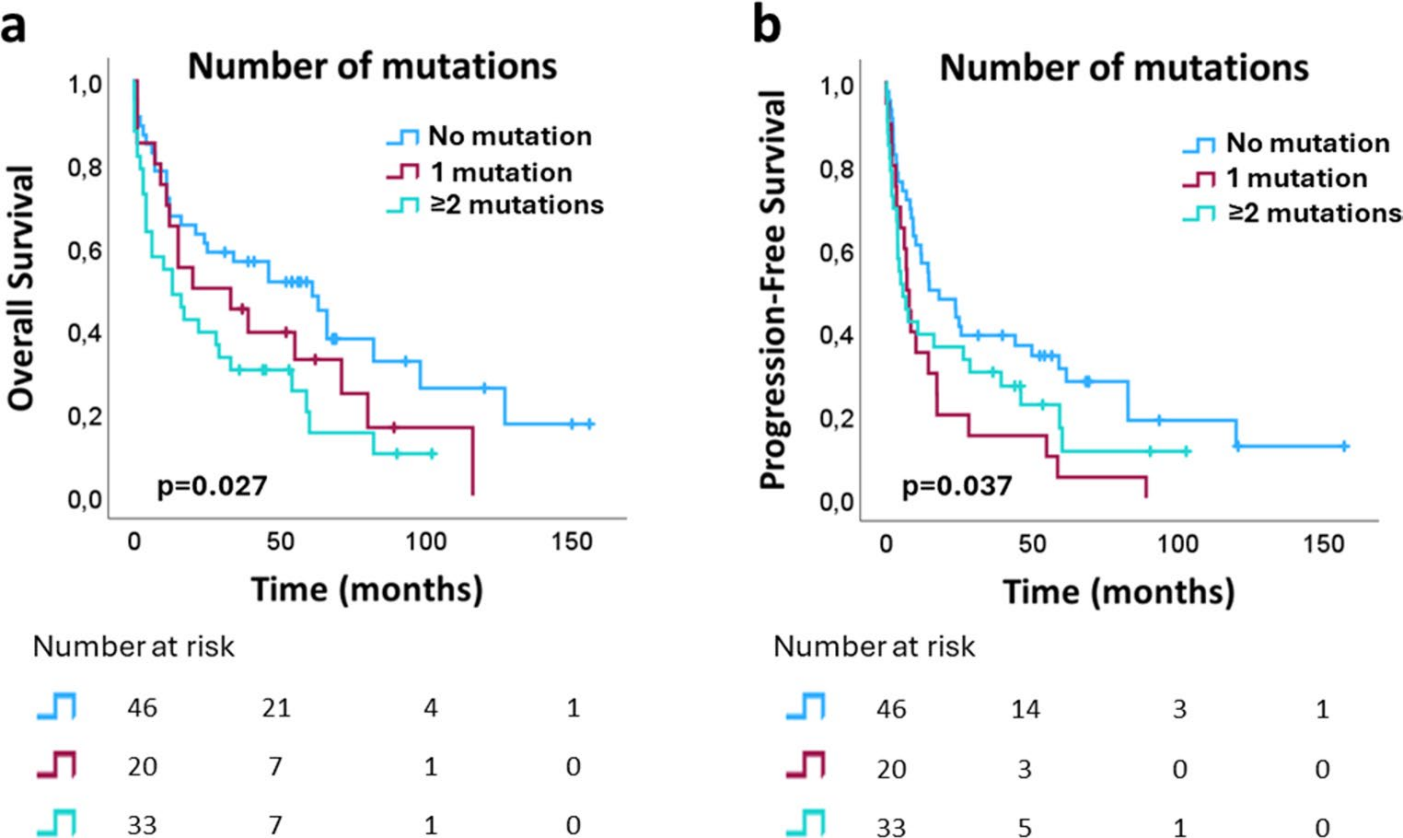
Kaplan-Meier plot (univariate analysis) of nTCL patients, demonstrating the influence of the number of mutations (pathogenic and likely pathogenic variants) on (**a**) overall survival and (**b**) progression-free survival

The median follow up time was 23 months (range: 0–156 months). At the end of the follow up period, 81 of 108 (75%) patients had died, 69 due to lymphoma. Univariate analysis identified several clinical characteristics associated with OS (Table 2; Supplementary Materials, Fig.S1). However, no significant association was found between individual mutations (P or LP variants) in *TET2*, *RHOA*, or *IDH2* and OS or PFS (Supplementary Materials, Fig.S2 and Fig.S4). Patients with co-mutations *TET2/RHOA* or *TET2/IDH2* showed no difference in OS or PFS compared to those with *TET2* mutations alone (Supplementary Materials, Fig.S3 and Fig.S5). Additional analysis revealed that the cumulative number of mutations significantly affected OS and PFS (*p* = 0.027 and *p* = 0.037), with differences observed across groups stratified by mutation count (Fig. 4). Multivariate analysis (Table 3) demonstrated that a high IPI score (4–5) (*p* = 0.001) and presence of ≥ 2 mutations (LP and P variants) (*p* = 0.013) are independent adverse prognostic factors for OS. Patients in the high-risk IPI group had a 5.832-fold increased risk of death (95% CI 1.964–17.315) compared to those in the low-risk group. Similarly, patients with ≥ 2 mutations had a higher risk of death (HR 2.232, 95% CI 1.182–4.214) compared to those without mutations. For PFS, however, only a high IPI score remained significant (*p* = 0.010; HR 3.429, 95% CI 1.351–8.703), whereas the presence of ≥ 2 mutations did not reach statistical significance (*p* = 0.191; HR 1.481, 95% CI 0.821–2.671).

## Discussion

This study investigated the mutational landscape of nTCL in a Slovenian cohort, providing additional data on known mutations and emphasizing the prognostic value of frequently mutated genes. Our results align with previous studies [4, 5, 8, 9, 12], confirming a similar mutation profile for nTFHL-AI and other nTFHL subtypes. Differences in OS were observed based on the number of mutations, response rates to first-line treatment, ASCT consolidation, LDH levels, and stratification by ECOG PS or IPI risk groups. Similar patterns were observed for PFS, except for ASCT consolidation.

For the first time, we report that nTCL patients with ≥ 2 mutations (in the same or different genes) have a higher risk of death compared to those with no mutations. This establishes the number of mutations as an independent adverse prognostic factor for OS alongside a high IPI score, PD after first-line treatment, or the absence of ASCT consolidation.

We observed frequent mutations in *TET2* (43%), *RHOA* (26%), *IDH2* (9%), *PLCG1* (8%), and *DNMT3A* (6%), all present exclusively in nTFHL. Notably, *TET2* was affected by multiple mutations in 42% of cases, consistent with numerous publications [4, 9, 10, 21, 24, 42–45]. Association analysis confirmed significant co-occurrence of *RHOA* mutations with *IDH2*, *DNMT3A*, and *TET2* mutations, as well as between *TET2* and *IDH2* [4, 9–12, 24, 46]. We verified that *TET2*, *RHOA*, and *DNMT3A* mutations are more frequent in nTFHL-AI (50%, 34%, and 4%) than in other nTFHL subtypes (24%, 18%, and 18%). In contrast, *TET2* mutations were more common in CL (63%), consistent with *TET2’*s role in B-cell lymphomagenesis in the context of nTFHL [47]. None of these mutations were detected in the 6 PTCL-NOS cases.

Lemonnier et al. also reported higher *TET2* mutation rates (47%) in AITL (now re-labelled to nTFHL-AI) compared to PTCL-NOS (38%) [21]. However, the PTCL-NOS group included cases now classified as nTFHL-NOS and nTFHL-F. Manso et al. found similar mutation frequencies in AITL and PTCL-NOS cohorts: *RHOA* (23.5%), *PLCG1* (14.3%), *IDH2* (11.2%), and *DNMT3A* (7.1%), but a lower *TET2* mutation rate (23.5%) than in our study [12]. Yao et al. reported higher mutation frequencies in *TET2*, *RHOA*, and *DNMT3A* in AITL (72%, 61%, and 34%) and PTCL-TFH (73%, 45%, and 36%) than in our study [9]. They observed multiple *TET2* mutations (≥ 2) in 57% of AITL and PTCL-TFH cases; similar to our finding that nearly half of *TET2*-mutated cases had multiple mutations. Dobay et al. reported a comparable *TET2* mutation rate (48%) but higher *RHOA* (58%) and *DNMT3A* (30%) mutation rates in AITL [5]. Vallois et al. found that *RHOA* mutations (60%) occurred more frequently than *TET2* mutations (52%) in TFH-derived PTCL [4]. In contrast to our results, Dobay et al. observed higher *TET2* mutation rates in TFH-derived PTCL (64%) and F-PTCL (75%) than in AITL (48%) [5].

All *IDH2* mutations (*n* = 9) in our study were R172X variants, consistent with other studies [9, 12]. These mutations were exclusive to nTFHL-AI, confirming their subtype specificity as previously reported [8, 11, 24]. The *IDH2*^R172X^ mutations were present in 12.3% of nTFHL-AI cases in our study, similar to the 11.2% reported by Manso et al. [12], though higher percentages were reported in other studies [4, 5, 10, 15, 24, 48, 49]. We also observed a co-occurrence of *IDH2*^R172X^ with *RHOA*^G17V^ mutations, in line with previous literature [8, 9, 12, 46].

Most *RHOA* mutations detected in our study were G17V (23 out of 26; 88.5%), consistent with previous reports [5, 9, 11, 24]. In addition, we detected three other *RHOA* mutations: E47K, R5Q, and R5W. In our cohort, only one *TP53* mutation was detected, occurring in an nTFHL-AI patient, similar to Ye et al. [24]. Unlike their findings, we did not observe *TP53* mutations in PTCL-NOS, likely due to the small number of respective cases in our study.

Overall, the mutational profile of Slovenian patients closely appears to match those reported in Western European and Asian cohorts, suggesting that genetic mechanisms driving nTFHL lymphomagenesis are tumour-specific rather than population-specific.

The adverse impact of *TET2*, *RHOA*, and *DNMT3A* mutations on survival outcomes in nTFHL has been documented in several studies [21–27]. These studies observed shorter PFS in patients carrying mutations in some of these genes, but no effect of individual mutations on OS. Consistent with these findings, our study found no significant association between individual *TET2*, *RHOA*, or *IDH2* mutations and OS. In contrast to some previous studies, we did not observe any association between these mutations and PFS [21, 27]. Similarly, Ye et al. observed no association between individual mutations and OS or PFS [24]. Only Tyler et al. found that *DNMT3A* mutations were linked to worse OS in a PTCL cohort, specifically in the PTCL-TBX21 molecular subtype [22]. Lemonnier et al. observed only a trend toward shorter PFS in patients with *TET2* mutations [21], while Hsu et al. reported significantly poorer PFS in patients with *RHOA* G17V-mutated AITL [27]. Additionally, we did not observe difference in OS or PFS between patients with *TET2/RHOA* or *TET2/IDH2* co-mutations compared to those with *TET2* mutations alone, corroborating Ye et al.‘s conclusion that these co-mutations do not impact OS [24]. However, Ye et al. showed that patients carrying *TET2*/*IDH2* co-mutations had better PFS compared to those with *TET2* mutations alone [24]. In line with previous reports [12], a considerable proportion of nTFHL patients in our study (22/99; 22.2%) had no detectable mutations. This variability prompted us to investigate whether the accumulation of mutations affects OS and PFS. We found a significant difference in OS and PFS across patient groups stratified by mutation count in univariate analysis. Multivariate analysis for OS confirmed that patients with ≥ 2 mutations (P or LP variants) had a 2.232-fold higher risk of death (95% CI 1.182–4.214) compared to those without mutations. Meanwhile, for PFS no statistical significance was observed (*p* = 0.191; HR 1.481 (0.821–2.671) but significance was confirmed in patients with a single mutation (*p* = 0.012; HR 2.351 (1.207–4.580). This may be due to the small size of the respective subgroups, which reduces the statistical power of the analysis. To our knowledge, this is the first study to identify the presence of ≥ 2 mutations as a new independent adverse prognostic factor for OS in nTCL, especially nTFHL.

In our cohort, CD10 expression was associated with *TET2* mutations, consistent with findings by Manso et al. [12]. Analysis of clinical parameters showed no significant association with either *TET2* or *RHOA* mutations. Although *TET2* mutations tended to correlate with B-symptoms, this did not remain significant after Bonferroni correction for multiple comparisons. Similarly, Manso et al. reported no association between these mutations and aggressive clinical features [12].

Most nTCL patients (including nTFHL-AI, PTCL-NOS, and ALCL) typically receive standard first-line therapies such as CHOP or CHOP-like regimens [21, 24, 27, 50, 51]. However, in our study, 57% received COP/COP-like or lower-dose treatments, achieving an ORR of 51.6% (27.4% CR, 24.2% PR). Additionally, 19% received CHOP/CHOP-like regimens, with an ORR of 65% (40% CR, 25% PR), consistent with prior studies [24, 52, 53]. Several studies have reported improved OS following ASCT consolidation therapy [24, 54, 55]. Indeed, in our cohort, patients who underwent ASCT consolidation showed prolonged OS, but not PFS. This may be due to the small size of the respective subgroups, reducing the statistical power of the analysis. There was also a significant OS difference according to response rates to first-line treatment. Multivariate analysis indicated that patients with PD after first-line treatment had a 7.682-fold higher risk of death compared to those achieving CR.

The present study shows for the first time that the presence of ≥ 2 mutations in nTCL, especially nTFHL, negatively impacts OS and serves as an independent prognostic factor alongside IPI, PD after first-line treatment, and ASCT consolidation. The mutational landscape of Slovenian nTFHL patients mirrors findings from previous studies, with frequent mutations in *TET2*,* RHOA*,* IDH2*,* DNMT3A*, and *PLCG1*, and notable co-occurrence among some of these genes. Additionally, our results highlight the importance of genetic profiling in routine PTCL diagnostics and prognostication. Such profiling may guide tailored treatment strategies, including predicting sensitivities to hypomethylating agents in *DNMT3A* and/or *TET2* mutated cases or IDH inhibitors in *IDH2*-mutated patients.

## Supplementary Information

Below is the link to the electronic supplementary material.

Supplementary Material 1 (DOCX 678 KB)

## Data Availability

The sequencing datasets generated and analysed during the study have been deposited in the NCBI (GenBank) under the project accession number PRJNA1225300 (Submission ID: SUB15071010).
