# Supplementary material for: Insights into Nodal T-Follicular Helper Cell lymphomas and Peripheral T-Cell Lymphomas, Not Otherwise Specified, in Slovenian Patients: Mutational Landscape, Clinicopathological Characteristics, and Outcomes
Source: Ann Hematol. 2025 Nov 13;104(12):6237–49. doi: 10.1007/s00277-025-06709-z (PMC12764645; doi:10.1007/s00277-025-06709-z)
Supplement: Supplementary file 1 — Supplementary Material 1 (DOCX 678 KB) [file 277_2025_6709_MOESM1_ESM.docx]

**Insights into Nodal T-Follicular Helper Cell Lymphomas and Peripheral T-Cell Lymphomas, Not Otherwise Specified, in Slovenian Patients:** **Mutational Landscape, Clinicopathological Characteristics, and Outcomes**

*Annals of Hematology*

Eva Erzar^1, 2^, Vanesa Sindi-Ivanova^3^, Stefan Dirnhofer^3^, Lučka Boltežar^2,4^, Janja Ocvirk^2,4^, Veronika Kloboves Prevodnik^1, 5^, Alexandar Tzankov^3†*^, Gorana Gašljević^5,6†*^

^1^ Department of Cytopathology, Institute of Oncology Ljubljana, Ljubljana, Slovenia

^2^ Faculty of Medicine, University of Ljubljana, Ljubljana, Slovenia

^3^ Pathology, Institute of Medical Genetics and Pathology, University Hospital Basel, University of Basel, Switzerland

^4^ Department of Medical Oncology, Institute of Oncology Ljubljana, Ljubljana, Slovenia

^5^ Faculty of Medicine, University of Maribor, Maribor, Slovenia

^6^ Department of Pathology, Institute of Oncology Ljubljana, Ljubljana, Slovenia

^†^ Equal contribution and shared last authorship.

**^*^Correspondence:** [alexandar.tzankov@usb.ch](mailto:alexandar.tzankov@usb.ch) (https://orcid.org/0000-0002-1100-3819) and [ggasljevic@onko-i.si](mailto:ggasljevic@onko-i.si) (https://orcid.org/0000-0002-8332-3627)

**SUPPLEMENTARY RESULTS**

**Table S1.** Panel of antibodies for IHC staining.

| **Antigen** | **Provider** | **Clone** | **Visualization** | **Antigen retrieval** | **Dilution** | **Incubation (min.)** |
| --- | --- | --- | --- | --- | --- | --- |
| **Bcl-6** | Cell Marque | GI191E/A8 | OptiView | CC1 | 1:800 | 60 |
| **CD2** | Epitomics | EP222 | OptiView | CC1 | 1:100 | 60 |
| **CD3** | Dako | polyclonal | OptiView | CC1 | 1:400 | 60 |
| **CD4** | Cell Marque | SP35 | OptiView | CC1 | 1:10 | 32 |
| **CD5** | Novocastra | 4C7 | OptiView | CC1 | 1:400 | 60 |
| **CD7** | Cell Marque | MRQ-56 | OptiView | CC1 | 1:200 | 60 |
| **CD8** | Dako | C8/144B | OptiView | CC1 | 1:100 | 60 |
| **CD10** | Novocastra | 56C6 | OptiView | CC1 | 1:20 | 60 |
| **CD20** | Dako | L26 | OptiView | CC1 | 1:500 | 60 |
| **CD21** | Cell Marque | EP3093 | OptiView | CC1 | 1:200 | 60 |
| **CD23** | Cell Marque | 1B12 | OptiView | CC1 | 1:50 | 32 |
| **CD30** | Cell Marque | Ber-H2 | OptiView | CC1 | 1:50 | 60 |
| **CD56** | Cell Marque | MRQ-42 | OptiView | CC1 | 1:200 | 60 |
| **CD278 (ICOS)** | Abcam | SP98 | OptiView | CC1 | 1:50 | 60 |
| **CXCL13** | RD systems | 53602 | OptiView | P1 | 1:50 | 60 |
| **GATA3** | Cell Marque | L50-823 | OptiView | CC1 | 1:400 | 60 |
| **GranB** | Dako | GrB-7 | OptiView | CC1 | 1:10 | 60 |
| **Ki-67** | Dako | MIB-1 | OptiView | CC1 | 1:200 | 60 |
| **p53** | Dako | D0-7 | OptiView | CC1 | 1:3200 | 60 |
| **PD-1** | Cell Marque | MRQ-22 | OptiView | CC1 | 1:800 | 60 |
| **Perforin** | Cell Marque | MRQ-23 | OptiView | CC1 | 1:50 | 60 |
| **TCRβ** | Invitrogen | 8A3 | OptiView | P1 | 1:100 | 60 |
| **TCRδ** | Santa Cruz Biot. | H-41 | OptiView | CC1 | 1:50 | 60 |
| **TIA-1** | Beckman Coulter | 2G9A10F5 | OptiView | CC1 | 1:2000 | 60 |

**Table S2.** Presentation of IHC staining for TFH markers and specific mutational findings for three re-classified samples (P53, P93, and P105).

|  |  |  | ***TFH markers*** | | | | |  | | |
| --- | --- | --- | --- | --- | --- | --- | --- | --- | --- | --- |
| **Patient ID** | **Initial Diagnosis** | **Revised Diagnosis** | **CD10** | **Bcl-6** | **PD-1** | **ICOS** | **CXCL13** | **Present mutations** | | |
| **P53** | PTCL-NOS  (T zone) | nTFHL-F | 0 | 0 | 5 | 3 | 0 | *DNMT3A* | *RHOA* |  |
| **P93** | PTCL-NOS (Lennert lymphoma) | nTFHL-NOS | 0 | 0 | 0 | 3 | 3 | *CREBBP* | *DNMT3A* | *RHOA* |
| **P105** | PTCL-NOS (Lennert lymphoma) | nTFHL-NOS | 0 | 5 | 6 | / | / | *B2M* | *KMT2D* |  |

**Expression Intensity Legend**: 0 = Negative; 1 = Weakly partially negative (more -ve than +ve); 2 = Weakly partially positive (more +ve than -ve); 3 = Partially positive (more -ve than +ve); 4 = Partially positive (more +ve than -ve); 5 = Weakly positive; 6 - Positive; 7 - Overexpression.

**Table S3.** Mutational repertoire showing all distinct pathogenic and likely pathogenic mutations, as well as variants of unknown significance, identified by targeted NGS in the series of nTCL cases (Excel file titled "Supplementary Material_Table S3").

**Table S4.** Results of the Pearson’s Chi-square or Fisher’s exact tests for association between TFH markers and *TET2* or *RHOA* mutations in the nTCL cohort.

|  | **Total (N)** | ***TET2*** | **p-value** | ***Phi Coefficient (Φ)* [Approx. Sig.]** | ***RHOA*** | **p-value** | ***Phi Coefficient (Φ)* [Approx. Sig.]** |
| --- | --- | --- | --- | --- | --- | --- | --- |
| **Bcl-6** | 96 | 33 | 0.284 | 0.109 [0.284] | 22 | 0.081 | 0.178 [0.081] |
| **CD10** | 96 | 22 | **0.003*** | 0.309 [0.003] | 14 | 0.010 | 0.262 [0.010] |
| **CXCL13** | 81 | 5 | 0.735 | 0.061 [0.583] | 5 | 0.049 | 0.235 [0.034] |
| **ICOS** | 89 | 25 | 0.493 | -0.073 [0.493] | 16 | 0.719 | 0.038 [0.719] |
| **PD-1** | 99 | 42 | 0.073 | 0.206 [0.040] | 24 | 1.000 | 0.029 [0.773] |

**Table S5**. Results of the Pearson’s Chi-square or Fisher’s exact tests for association between clinical characteristics and *TET2* or *RHOA* mutational status in the nTCL cohort.

| **Charateristics** | **WT *TET2*** | **MUT *TET2*** | **p-value** | **WT *RHOA*** | **MUT *RHOA*** | **p-value** |
| --- | --- | --- | --- | --- | --- | --- |
| **Number of patients** | 56 | 43 |  | 73 | 26 |  |
| **Diagnosis (lymphoma subtype)** |  |  | 0.045 |  |  | 0.100 |
| nTFHL-AI | 34/68 | 34/68 |  | 45/68 | 23/68 |  |
| nTFHL-NOS | 10/13 | 3/13 |  | 11/13 | 2/13 |  |
| nTFHL-F | 3/4 | 1/4 |  | 3/4 | 1/4 |  |
| CL | 3/8 | 5/8 |  | 8/8 | 0/8 |  |
| PTCL-NOS | 6/6 | 0/6 |  | 6/6 | 0/6 |  |
| **Gender** |  |  | 0.678 |  |  | 0.970 |
| Male | 31/53 | 22/53 |  | 39/53 | 14/53 |  |
| Female | 25/46 | 21/46 |  | 34/46 | 12/46 |  |
| **Age at diagnosis** |  |  | 0.293 |  |  | 0.774 |
| >60 years | 42/78 | 36/78 |  | 57/78 | 21/78 |  |
| ≤60 years | 14/21 | 7/21 |  | 16/21 | 5/21 |  |
| **Ann Arbor stage** |  |  |  |  |  | 1.000 |
| I-II | 3/8 | 5/8 | 0.289 | 6/8 | 2/8 |  |
| III-IV | 53/91 | 38/91 |  | 67/91 | 24/91 |  |
| **B-symptoms** |  |  | 0.034 |  |  | 0.436 |
| No | 23/32 | 9/32 |  | 22/32 | 10/32 |  |
| Yes | 33/67 | 34/67 |  | 51/67 | 16/67 |  |
| **Raised serum LDH level** |  |  | 0.805 |  |  | 0.758 |
| No | 26/44 | 18/44 |  | 32/44 | 12/44 |  |
| Yes | 30/53 | 23/53 |  | 40/53 | 13/53 |  |
| **ECOG performance status** |  |  | 0.181 |  |  | 0.577 |
| 0-1 | 36/69 | 33/69 |  | 52/69 | 17/69 |  |
| 2-4 | 20/30 | 10/30 |  | 21/30 | 9/30 |  |
| **IPI** |  |  | 0.438 |  |  | 0.499 |
| Low-risk group | 8/13 | 5/13 |  | 9/13 | 4/13 |  |
| Low-intermediate-risk group | 13/22 | 9/22 |  | 19/22 | 3/21 |  |
| High-intermediate-risk group | 19/39 | 20/39 |  | 27/39 | 12/39 |  |
| High-risk group | 16/23 | 7/23 |  | 17/23 | 6/23 |  |

**
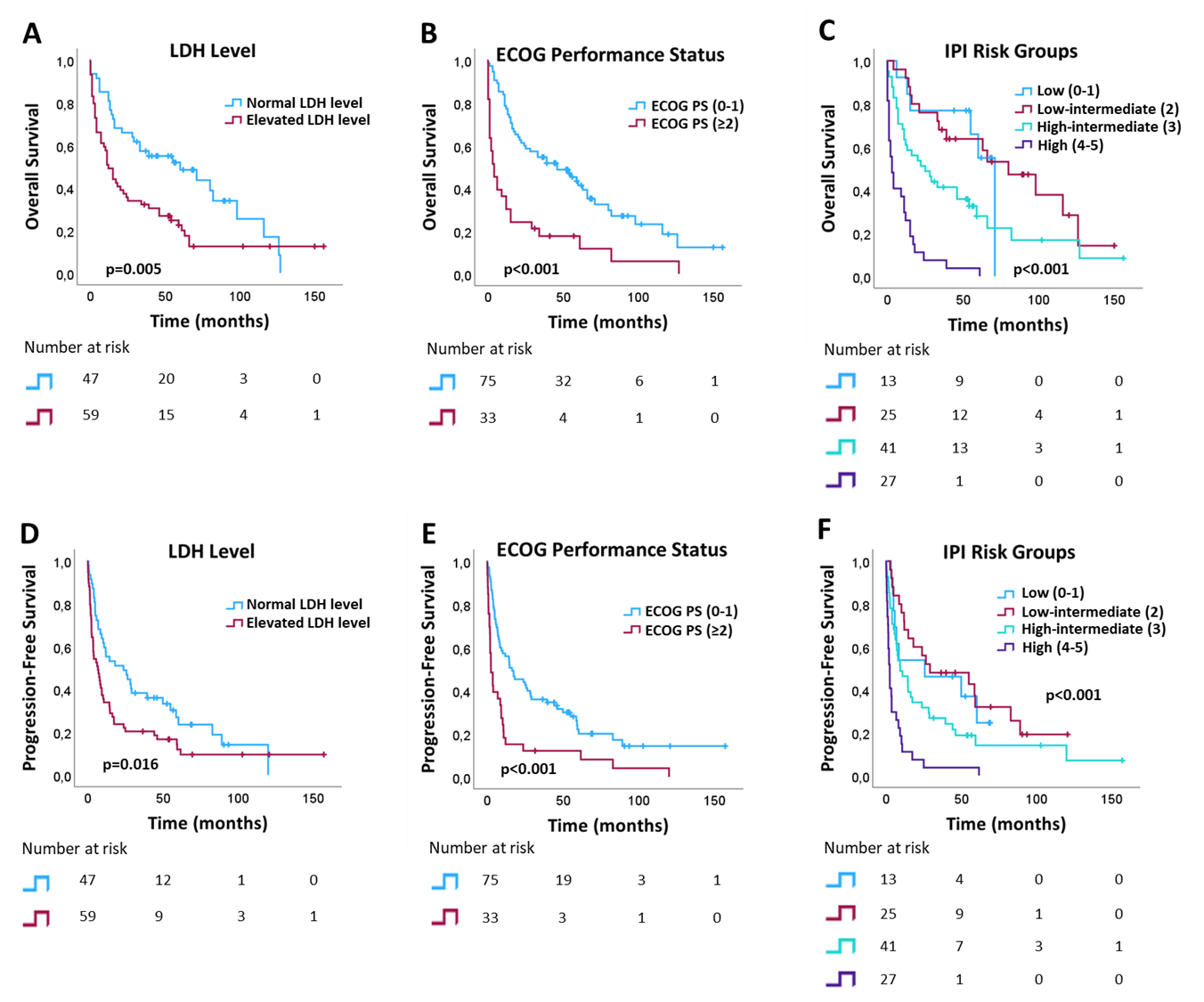
**

**Fig.S1** Kaplan-Meier plots (univariate analysis) for overall survival and progression-free survival, showing only factors with significant differences: (A, D) LDH level, (B, E) ECOG performance status (PS), and (C, F) IPI risk groups, among all analyzed clinical characteristics of the nTCL patients.

LDH: lactate dehydrogenase; ECOG: Eastern Cooperative Oncology Group; IPI: International Prognostic Index.

**
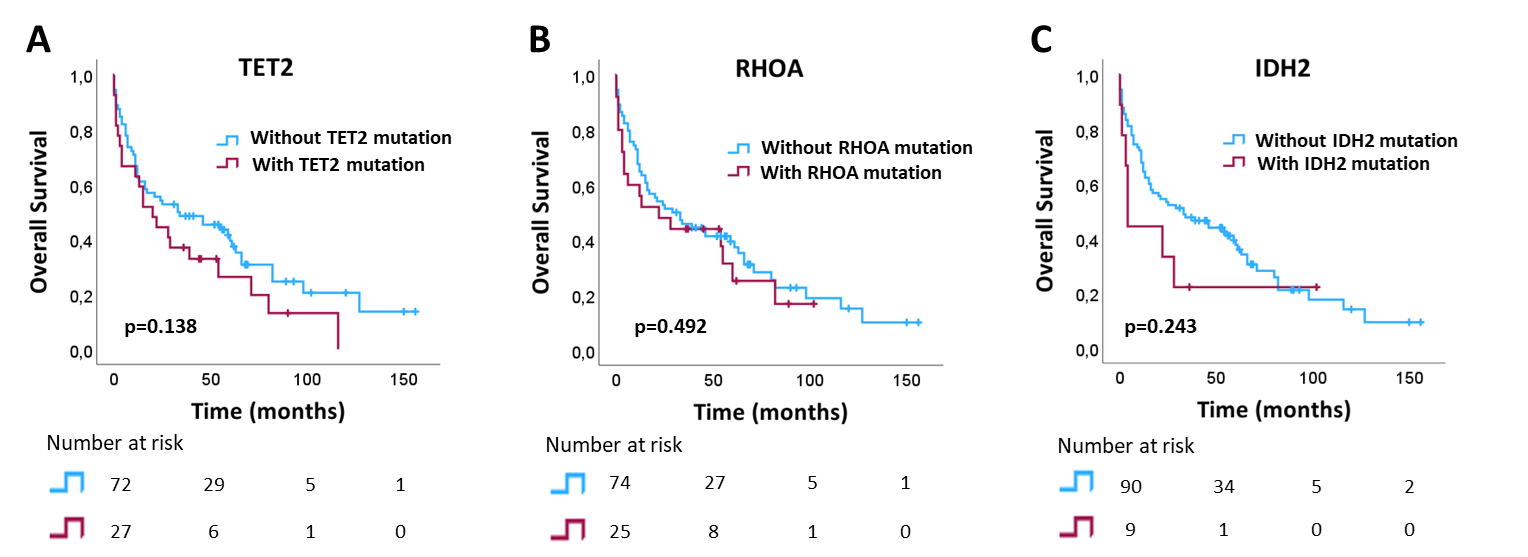
**

**Fig.S2** Kaplan-Meier plots (univariate analysis) of the nTCL patients, demonstrating the impact of the three most frequent mutations (A) *TET2*, (B) *RHOA,* and (C) *IDH2* on the overall survival.

*TET2*: Tet Methylcytosine Dioxygenase 2; *RHOA*: Ras Homolog Family Member A; *IDH2*: Isocitrate Dehydrogenase (NADP (+)) 2; ns: not significant.

**
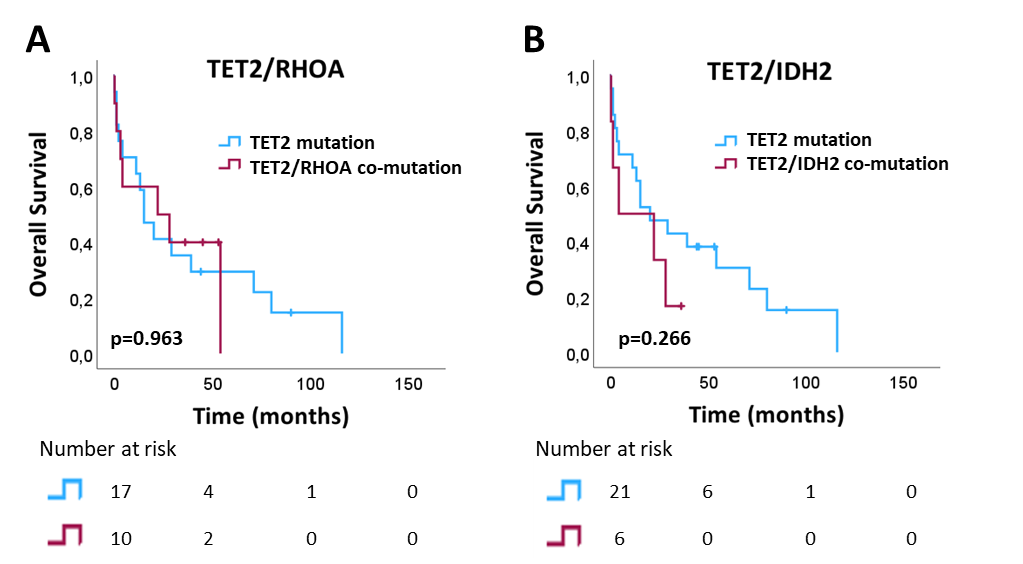
**

**Fig.S3** Kaplan-Meier plots (univariate analysis) of the nTCL patients, demonstrating the influence of (A) *TET2/RHOA* and (B) *TET2/IDH2* co-mutations on the overall survival.

*TET2*: Tet Methylcytosine Dioxygenase 2; *RHOA*: Ras Homolog Family Member A; *IDH2*: Isocitrate Dehydrogenase (NADP (+)) 2; ns: not significant.


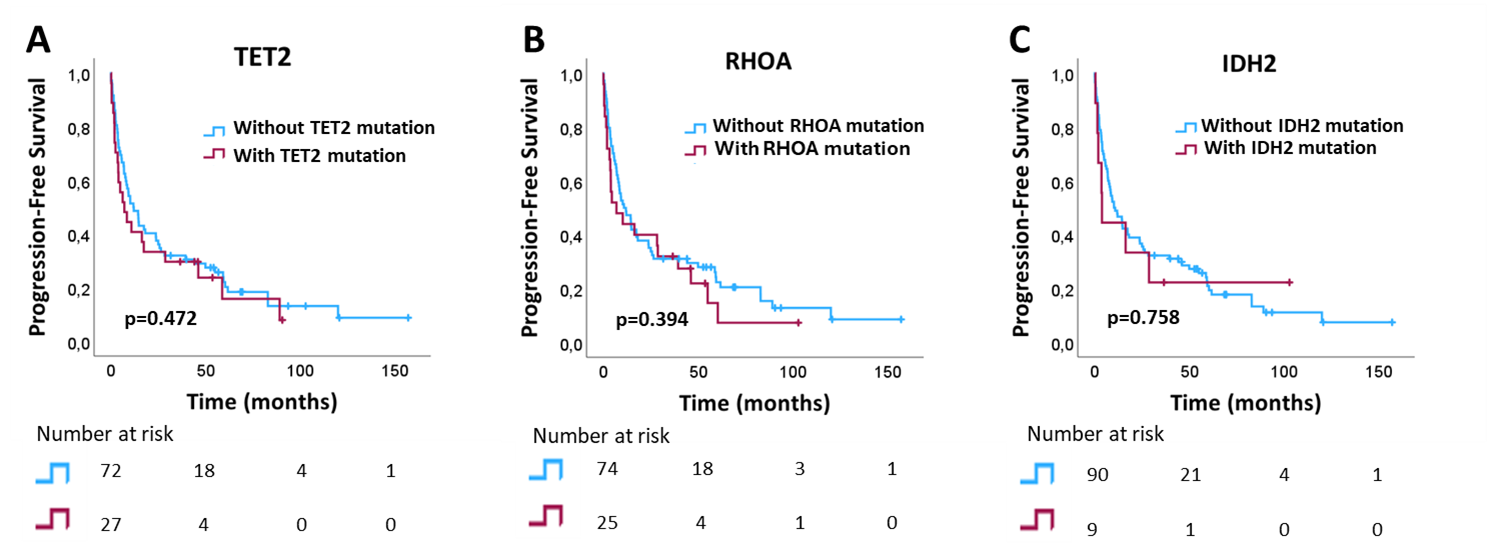


**Fig.S4** Kaplan-Meier plots (univariate analysis) of the nTCL patients, demonstrating the impact of the three most frequent mutations (A) TET2, (B) RHOA, and (C) IDH2 on the progression-free survival.

TET2: Tet Methylcytosine Dioxygenase 2; RHOA: Ras Homolog Family Member A; IDH2: Isocitrate Dehydrogenase (NADP (+)) 2; ns: not significant.


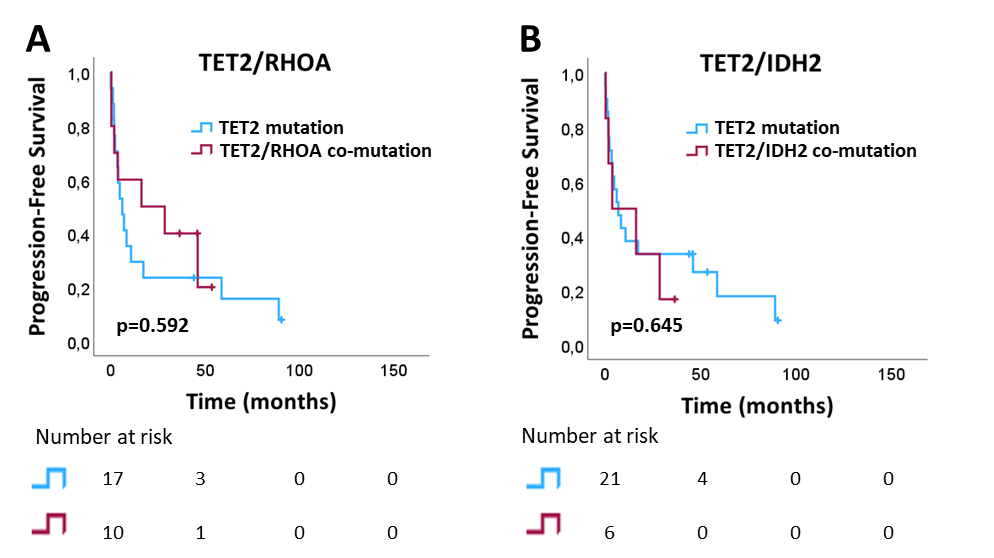


**Fig.S5** Kaplan-Meier plots (univariate analysis) of the nTCL patients, demonstrating the influence of (A) *TET2/RHOA* and (B) *TET2/IDH2* co-mutations on the progression-free survival.

*TET2*: Tet Methylcytosine Dioxygenase 2; *RHOA*: Ras Homolog Family Member A; *IDH2*: Isocitrate Dehydrogenase (NADP (+)) 2; ns: not significant.
